# Emicizumab, A Bispecific Antibody to Factors IX/IXa and X/Xa, Does Not Interfere with Antithrombin or TFPI Activity In Vitro

**DOI:** 10.1055/s-0038-1636538

**Published:** 2018-03-16

**Authors:** Mariko Noguchi-Sasaki, Tetsuhiro Soeda, Atsunori Ueyama, Atsushi Muto, Michinori Hirata, Hidetomo Kitamura, Kaori Fujimoto-Ouchi, Yoshiki Kawabe, Keiji Nogami, Midori Shima, Takehisa Kitazawa

**Affiliations:** 1 Medical Affairs Division, Chugai Pharmaceutical Co., Ltd., Kamakura, Kanagawa, Japan; 2 Research Division, Chugai Pharmaceutical Co., Ltd., Gotemba, Shizuoka, Japan; 3 Department of Pediatrics, Nara Medical University, Kashihara, Nara, Japan

**Keywords:** emicizumab, antithrombin, TFPI, coagulation factors, hemophilia A

## Abstract

Emicizumab is a humanized bispecific antibody that binds simultaneously to factor (F) IXa and FX replacing the cofactor function of FVIIIa. Because emicizumab recognizes FIX/FIXa and FX/FXa, a question may arise whether emicizumab competes with antithrombin (AT) and/or tissue factor pathway inhibitor (TFPI), thereby enhancing overall hemostatic potential by blocking their antihemostatic effects. To address this question, we performed enzymatic assays using purified coagulation factors to confirm whether emicizumab interferes with the action of AT on FIXa or FXa, or with the action of TFPI on FXa. In those assays, we found no interference of emicizumab on the actions of AT and TFPI. We next assessed emicizumab's influences on the anticoagulation actions of AT or TFPI in thrombin generation assays triggered with FXIa or tissue factor (TF) in AT-depleted or TFPI-depleted plasma supplemented with AT or TFPI in vitro. In those assays, we employed anti-FIXa and anti-FX monospecific one-armed antibodies derived from emicizumab instead of emicizumab itself so as to prevent emicizumab's FVIIIa cofactor activity from boosting thrombin generation. Consequently, we found that neither anti-FIXa, anti-FX monospecific antibody, nor the mixture of the two interfered with the anticoagulation actions of AT or TFPI in plasma. Although emicizumab can bind to FIXa and FXa, our results showed no interference of emicizumab with the action of AT or TFPI on FIXa or FXa. This indicates that the presence of emicizumab is irrelevant to the action of AT and TFPI, and thus should not alter the coagulant/anticoagulant balance related to AT and TFPI.

## Introduction

Emicizumab (also known as ACE910) is an asymmetric humanized bispecific antibody that bridges factors (F) IXa and FX, accelerating FIXa-catalyzed FX activation and facilitating thrombin burst in FVIII-deficient plasma. 1 2 3 One mechanism of the cofactor function of FVIIIa is to maintain FIXa and FX in the appropriate positional relationship in the enzyme reaction on activated phospholipid membranes (e.g., activated platelet membrane at hemostatic site) on which FIXa activates FX. Emicizumab mimics the FVIIIa cofactor function as a kind of scaffold by binding and placing FIXa and FX into spatially appropriate positions. We previously demonstrated in nonhuman primate models of acquired hemophilia A that emicizumab exerted a hemostatic activity against ongoing bleeds artificially induced in muscles and subcutis 3 and prevented spontaneous joint bleeds. 4 In a Phase I clinical trial, prophylactic treatment with weekly subcutaneous administration of emicizumab was well tolerated and decreased the number of bleeding episodes in severe hemophilia A patients with or without FVIII inhibitors. 5 A Phase III multicenter trial showed that emicizumab prophylaxis was associated with a significantly lower rate of bleeding events than no prophylaxis or previous prophylactic treatment with bypassing agents among patients with hemophilia A with FVIII inhibitors, and it improved health-related quality of life. 6 Emicizumab not only showed efficacy in preventing bleeding but also would overcome patients' and their caregivers' distress accompanied by frequent venous access for implementing prophylactic treatment with a FVIII or a bypassing agent. In addition, emicizumab is not expected to induce FVIII inhibitors as its molecular structure is totally different from that of FVIII.

Regulating negative control of the coagulation process is a new approach to the treatment of hemophilia A. Approaches recently being tried in clinical studies include therapeutic RNA interference (RNAi) targeting antithrombin (AT) 7 and anti-tissue factor pathway inhibitor (TFPI) antibodies. 8 9 10 AT is a serine protease inhibitor (serpin) synthesized in the liver that physiologically inactivates thrombin and FXa, and to a lesser extent FIXa, FXIa, FXIIa, and other procoagulant factors, when the active reactive center of AT binds to the catalytic sites of those coagulation factors. 11 TFPI is a Kunitz-type proteinase inhibitor that regulates the tissue factor (TF) pathway of coagulation initiation. 12 Kunitz domains 1 and 2 directly bind to and inhibit the active site of FVIIa and FXa, respectively. 12

Thus, emicizumab and AT can bind to FIXa, and emicizumab, AT, and TFPI can bind to FXa. This raises the question of whether emicizumab may interfere with the actions of AT or TFPI; in other words, whether emicizumab's mechanism of action would include the lowering of AT or TFPI activities. We anticipated that emicizumab would be unlikely to interfere with the actions of AT or TFPI, because emicizumab binds the epidermal growth factor (EGF)-like domains of FIXa and FXa, while AT and TFPI bind the protease domains, and because its binding affinities are on the order of 1 micromolar. 13 However, we thought it is very important to support by actual experimental data that emicizumab's mechanism of action be strictly clarified. In this study, we investigated whether emicizumab interferes with the action of AT on FIXa and FXa or with the action of TFPI on FXa by means of enzymatic assays, and whether emicizumab impedes the anticoagulation activities of AT and TFPI through plasma thrombin generation assays.

## Materials and Methods

### Materials

Emicizumab (recombinant humanized IgG _4_ ) was produced from a Chinese hamster ovary cell line using recombinant DNA technology as previously reported. 14 Plasma emicizumab concentrations around 10.0 to 100 μg/mL (or 68.7–687 nM) was shown to be clinically effective. 5 Anti-FIXa or anti-FX monospecific one-armed IgG _4_ antibodies having either of the Fabs of emicizumab were transiently expressed in HEK293 cells and purified. 1 FIXa, FXa, FXIa, and AT, all derived from human plasma, were purchased from Enzyme Research Laboratories (South Bend, Indiana, United States). TFPI (C-terminally truncated TFPI) was obtained from R&D Systems (Minneapolis, Minnesota, United States). AT-deficient and TFPI-deficient plasmas were obtained from Sekisui Diagnostics (Lexington, Massachusetts, United States). Phospholipids (Avanti Polar Lipids, Alabaster, Alabama, United States) consisting of 10% phosphatidylserine, 60% phosphatidylcholine, and 30% phosphatidylethanolamine were prepared as previously reported. 15 Heparin sodium (molecular weight: 5,000–20,000) was obtained from Nipro (Osaka, Japan).

### Chromogenic Assays for FIXa and FXa Activities

To evaluate FIXa inhibition, we conducted an enzymatic assay consisting of 89 nM human FIXa, 4 μM phospholipids, various concentrations of AT, and emicizumab in Tris-buffered saline (50 mM Tris, 150 mM NaCl) containing 1 mM CaCl _2_ and 0.1% (w/v) bovine serum albumin (BSA), and incubated for 30 minutes at room temperature. After adding a chromogenic substrate specific to FIXa (Spectrozyme FIXa; Sekisui Diagnostics), we measured absorbance at 405 nm to determine FIXa activity. We performed the experiment in the presence of 30 IU/mL heparin under the same conditions.

To evaluate FXa inhibition, we conducted an enzymatic assay consisting of 1.25 nM human FXa, 4 μM phospholipids, various concentrations of either AT or TFPI, and emicizumab in Tris-buffered saline containing 1 mM CaCl _2_ and 0.1% (w/v) BSA, and incubated for 30 minutes at room temperature. After adding a chromogenic substrate specific to FXa (S-2222; Sekisui Diagnostics), we measured absorbance at 405 nm to determine FXa activity. In the enzymatic assay containing AT, we performed the experiment in the presence of 0.005 IU/mL heparin under the same conditions.

### Thrombin Generation Assay

Thrombin generation assays were performed with standard equipment and calibrated automated thrombography (CT) using a 96-well plate fluorometer (Thermo Fisher Scientific, Waltham, Massachusetts, United States) equipped with analyzing software (Thrombinoscope BV, Maastricht, the Netherlands). Thrombin generation was initiated with an intrinsic pathway triggering solution containing 0.04 nM human FXIa and 20 μM phospholipids in Tris-buffered saline solution containing 0.1% (w/v) BSA, or extrinsic pathway triggering solutions (PPP-Reagent HIGH, PPP-Reagent LOW; Thrombinoscope BV) in AT- or TFPI-depleted plasma reconstituted with AT or TFPI. In the intrinsic pathway triggering solution, we adopted the FXIa concentration which can detect the changes of thrombin generation parameters sensitively around the plasma concentration of emicizumab (around 50 μg/mL = 343 nM) in the HAVEN1 Phase III study (see also Supplementary Fig. S6A ). To initiate the reaction, 20 μL of FluCa reagent prepared from a FluCa kit (Thrombinoscope BV) was dispensed by the instrument as programmed. We analyzed the thrombograms, peak height, lag time, and endogenous thrombin potential (ETP) by the instrument's software. To exclude the effect of emicizumab's FVIIIa cofactor function on coagulation, anti-FIXa and anti-FX monospecific one-armed antibodies derived from emicizumab were used instead of emicizumab in this assay.

## Results

### Enzymatic Assays to Assess the Effect of Emicizumab on the Action of AT on FIXa and FXa and on the Action of TFPI on FXa

We first determined the appropriate conditions for the chromogenic assays for FIXa and FXa activities with which to assess AT or TFPI inhibitory action on FIXa or FXa. In the assays, the measured absorbance increased in a FIXa- or FXa-concentration–dependent manner ( Supplementary Fig. S1 ). On the basis of those results, we adopted concentrations of 89 nM FIXa and 1.25 nM FXa which are not saturated and at which it would be possible to detect changes in FIXa or FXa activity in the next part of the experiments.

**Fig. 1:**
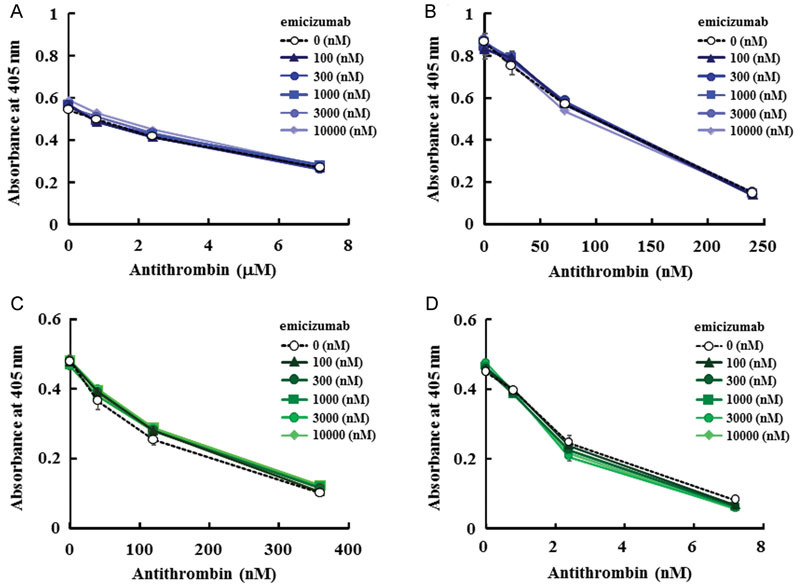
Effect of emicizumab on the action of AT on FIXa and FXa in enzymatic assays. ( **A** ) Emicizumab's effect on the action of AT on FIXa. ( **B** ) Emicizumab's effect on the action of AT on FIXa in the presence of heparin. ( **C** ) Emicizumab's effect on the action of AT on FXa. ( **D** ) Emicizumab's effect on the action of AT on FXa in the presence of heparin. Data are expressed as mean ± SD ( *n* = 3). The bars depicting SD are shorter than the height of the symbols. The symbols for the groups with higher concentrations of emicizumab are hidden behind the symbols for the other groups.

To assess the inhibitory action of AT on FIXa and FXa, and any potential modulation of this action by emicizumab, we performed the chromogenic assays for FIXa and FXa activities under the conditions mentioned earlier. Because the inhibitory effect of AT on FIXa and FXa is strongly enhanced by heparin, we evaluated the inhibitory effect of AT on FIXa and FXa with or without heparin. We confirmed that AT decreased FIXa activity dose dependently, and that its inhibitory effect was enhanced in the presence of heparin ( Fig. 1A , B ). Various concentrations of emicizumab did not affect the AT's action to inhibit FIXa activity in either the presence or absence of heparin ( Fig. 1A , B ). We also confirmed that AT decreased FXa activity dose dependently, and that its inhibitory effect was enhanced in the presence of heparin ( Fig. 1C , D ). Various concentrations of emicizumab did not affect the AT's action to inhibit FXa activity in either the presence or absence of heparin ( Fig. 1C , D ). Taken together, these results indicate that emicizumab did not interfere with the inhibitory activity of AT on FIXa or FXa.

**Fig. 2:**
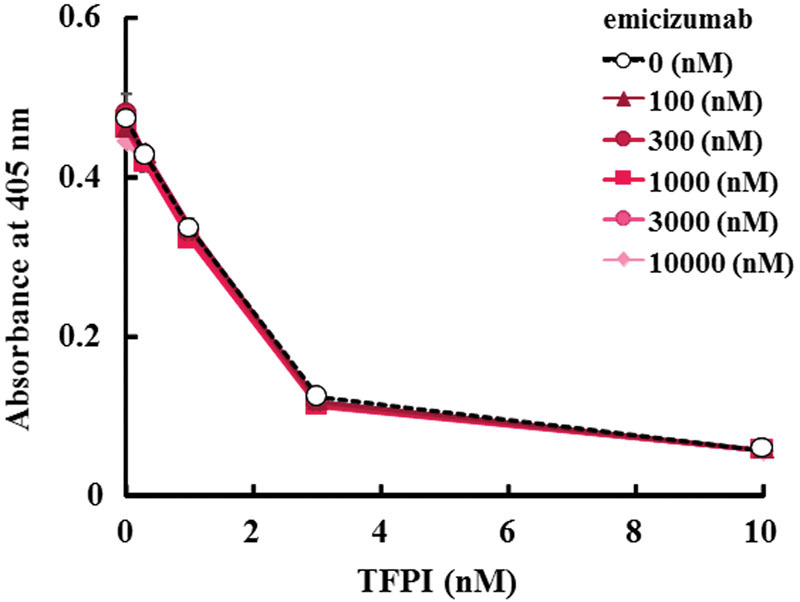
Effect of emicizumab on the action of TFPI on FXa in enzymatic assays. Emicizumab's effect on the action of TFPI on FXa in the FXa inhibition assay. Data are expressed as mean ± SD ( *n* = 3). The bars depicting SD are shorter than the height of the symbols. The symbols for the groups with higher concentrations of emicizumab are hidden behind the symbols for the other groups.

We next evaluated the effect of emicizumab on the inhibitory action of TFPI on FXa by using the chromogenic assay for FXa activity. We confirmed that C-terminus truncated TFPI decreased FXa activity dose dependently ( Fig. 2 ). Various concentrations of emicizumab did not affect the inhibition of FXa by C-terminus truncated TFPI ( Fig. 2 ). We also performed the effect of emicizumab on the action of full-length TFPI on FXa in enzymatic assays, and we confirmed that various concentrations of emicizumab did not inhibit full-length TFPI activity on FXa ( Supplementary Fig. S2 ).

### Effects of Anti-FIXa and Anti-FX Monospecific One-Armed Antibodies on Thrombin Generation in AT- or TFPI-Depleted Plasma

We demonstrated in the enzymatic assays that emicizumab did not interfere with the action of AT or TFPI on FIXa or FXa. To evaluate these effects in the presence of other coagulation-related factors, we next evaluated the effects of emicizumab on thrombin generation in AT- and TFPI-depleted plasma. As emicizumab does not need to be activated to exert FVIIIa cofactor function on coagulation, it has potential to affect parameters in thrombin generation. Because thrombin generation assays cannot distinguish between the potential blocking effects of emicizumab on AT or TFPI's action versus the effect of emicizumab's FVIIIa cofactor function, both of which would increase thrombin generation, we first had to prevent the effect of emicizumab's FVIIIa cofactor function on coagulation. To accomplish this, we generated anti-FIXa and anti-FX monospecific one-armed antibodies derived from emicizumab for use in the thrombin generation assays instead of emicizumab.

**Fig. 3:**
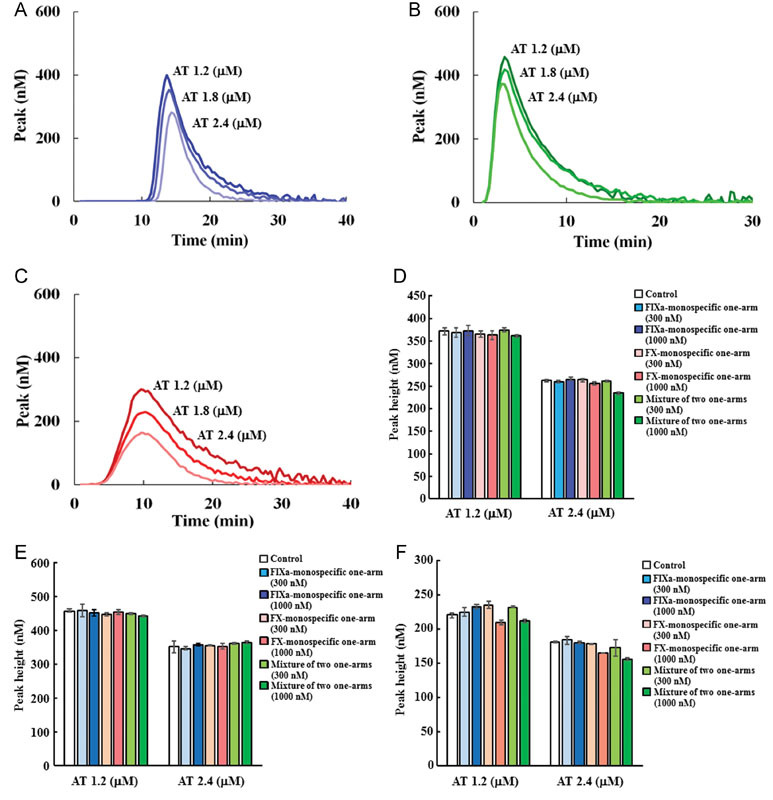
Effects of AT and FIXa and FX monospecific one-armed antibodies on thrombin generation in AT-depleted plasma. Effects of various concentrations of AT on the thrombin generation parameter peak height in AT-depleted plasma triggered by ( **A** ) FXIa, ( **B** ) high concentration of TF, and ( **C** ) low concentration of TF. Data were collected in triplicate and representative thrombograms are shown. Effects of various concentrations of anti-FIXa and anti-FX monospecific one-armed antibodies, and mixtures of the two one-armed antibodies on the thrombin generation parameter peak height in AT-depleted plasma triggered by ( **D** ) FXIa, ( **E** ) high concentration of TF, and ( **F** ) low concentration of TF in the presence of 1.2 or 2.4 μM of AT. Data are expressed as mean ± SD ( *n* = 3).

To assess the effects of anti-FIXa and anti-FX monospecific one-armed antibodies on thrombin generation in AT-depleted plasma, we first confirmed in AT-depleted plasma that AT dose dependently decreased thrombin peak height under three triggering conditions (FXIa, high TF concentration, and low TF concentration) to evaluate both intrinsic and extrinsic pathways (representative thrombograms are shown in Fig. 3A – C ). As standard plasma concentration of AT was reported as 2.4 μM, 16 we employed 1.2 or 2.4 μM as normal physiological concentration of AT in plasma. Because AT-depleted plasma without addition of any AT exhibited extremely short lag time and high thrombin peak height, the thrombogram parameters could not be analyzed by the CT system (data not shown); therefore, we evaluated anti-FIXa and anti-FX monospecific one-armed antibodies in the presence of various concentrations of AT ( Fig. 3D – F , Supplementary Fig. S3 ). Anti-FIXa monospecific one-armed antibody, anti-FX monospecific one-armed antibody, and their equimolar mixtures were evaluated in AT-depleted plasma reconstituted with AT. Though there were variations of the parameters, all of the one-armed antibodies or their mixtures showed almost the same thrombin peak height, lag time, and ETP compared with the antibody-absent control group under all of the triggering conditions, suggesting that anti-FIXa and anti-FX monospecific one-armed antibodies and their mixtures did not inhibit the activity of AT on thrombin generation ( Fig. 3D – F ).

**Fig. 4:**
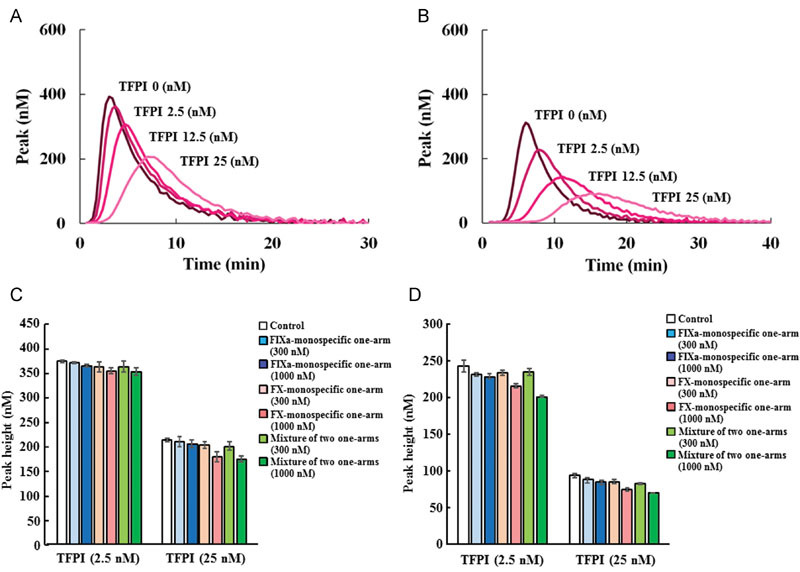
Effects of TFPI and anti-FIXa and anti-FX one-armed antibodies on thrombin generation in TFPI-depleted plasma. Effects of various concentrations of TFPI on the thrombin generation parameter peak height in TFPI-depleted plasma triggered by ( **A** ) high concentrations of TF and ( **B** ) low concentrations of TF. Data were collected in triplicate and representative thrombograms are shown. Effects of various concentrations of anti-FIXa and anti-FX monospecific one-armed antibodies, and mixtures of the two one-armed antibodies on the thrombin generation parameter peak height in TFPI-depleted plasma triggered by ( **C** ) high concentrations of TF and ( **D** ) low concentrations of TF in the presence of 2.5 and 25 nM of TFPI. Data are expressed as mean ± SD ( *n* = 3).

Finally, to assess the effects of anti-FIXa and anti-FX monospecific one-armed antibodies on thrombin generation in TFPI-depleted plasma, we confirmed in advance in TFPI-depleted plasma that TFPI dose dependently decreased thrombin peak height under two triggering conditions: high TF concentration and low TF concentration (representative thrombograms are shown in Fig. 4A , B ). As standard plasma concentration of TFPI was reported as 2.5 nM 16 and its concentration increases almost 10-fold during the activation of coagulation, we employed 2.5 or 25 nM as normal physiological concentration of TFPI in plasma. As with AT-depleted plasma, we evaluated anti-FIXa and anti-FX monospecific one-armed antibodies in the presence of various concentrations of TFPI ( Fig. 4C , D , Supplementary Fig. S4 ). Anti-FIXa monospecific one-armed antibody, anti-FX monospecific one-armed antibody, and their mixtures were evaluated in TFPI-depleted plasma reconstituted with TFPI. All of the one-armed antibodies and their mixtures did not increase the thrombin peak height and ETP, and did not shorten the lag time compared with the antibody-absent control group, suggesting that anti-FIXa and anti-FX monospecific one-armed antibodies did not inhibit the activity of TFPI on thrombin generation ( Fig. 4C , D ).

## Discussion

Coagulation factor replacement has become the current standard therapy for the treatment of hemophilia in developed countries. On the other hand, suppressor mechanisms are known to regulate coagulation, with AT and TFPI acting as two of the important inhibitors of coagulation in this feedback process; therefore, approaches that inhibit AT or TFPI are also expected to achieve hemostatic activity in bleeding disorders. A trial of humanized anti-TFPI monoclonal antibody (mAb 2021, concizumab) administered intravenously or subcutaneously to patients with hemophilia A and B showed a dose-dependent procoagulant effect. 8 An RNAi therapeutic drug that targets AT (fitusiran) was evaluated in patients with hemophilia A and B; weekly and monthly doses of fitusiran administered subcutaneously decreased AT levels and increased thrombin generation. 7 Evidence from these clinical trials indicates that AT and TFPI play important roles in regulating coagulation in hemophilia patients. In another newly developed approach to the treatment of hemophilia, we generated an anti-FIXa/FX bispecific antibody, emicizumab, which promotes FIXa-catalyzed FX activation. We have reported emicizumab's hemostatic activity and bleeding-preventive activities in animal and clinical studies. 3 4 5 6 Because emicizumab and AT can both bind to FIXa, and emicizumab, AT, and TFPI can each bind to FXa, the question arises as to whether emicizumab would interfere with the actions of AT or TFPI. In other words, whether emicizumab's mechanism of action would lessen the activities of AT or TFPI. Therefore, in this study, we investigated the effect of emicizumab on the action of AT or TFPI on FIXa or FXa, and we demonstrated by means of enzymatic assays and thrombin generation assays that emicizumab does not interfere with either the activity of AT on FIXa and FXa or the activity of TFPI on FXa. Our results indicated that emicizumab's hemostatic activity does not include AT or TFPI inhibition and, therefore, that emicizumab exerts its hemostatic activity at the step of FIXa-catalyzed FX activation via its FVIIIa cofactor function.

FIXa and FXa are inhibited by AT which binds to the active sites located on the heavy chain of these trypsin-like serine proteases, and FXa is inhibited by the Kunitz domain 2 of TFPI interacting with the serine protease domain located on the heavy chain of FXa. 12 On the other hand, emicizumab binds to the respective EGF-like domains located on the light chains of FIX/FIXa and FX/FXa. 1 2 13 It is reasonable to expect that differences in the binding sites on FIXa and FXa might be the reason why emicizumab does not interfere with the activity of AT and TFPI on these proteins. We also found that at least epitopes of emicizumab, EGF-like domain 1 of FIX/FIXa and EGF-like domain 2 of FX/FXa, do not affect interactions with AT or TFPI to inhibit these coagulation factors.

In this study, we demonstrated that activity of emicizumab does not include AT or TFPI inhibition, indicating that emicizumab does not interfere with coagulation feedback by AT or TFPI.

Emicizumab had little influence on thrombin peak height under three triggering conditions (FXIa, high TF concentration, low TF concentration) in normal plasma ( Supplementary Fig. S5 ). We think that this is because the thrombin peak height under these conditions does not have enough sensitivity to detect the add-on effect of emicizumab in the presence of normal level of FVIII. Emicizumab slightly extended lag time in high concentrations of emicizumab under high TF concentration and low TF concentration triggering condition, whereas emicizumab showed marked shortened lag time in FXIa triggering condition ( Supplementary Fig. S5 ). In the FVIII-deficient plasma, emicizumab increased thrombin peak height and ETP under FXIa and low TF concentration triggering conditions, shortened lag time under FXIa triggering condition ( Supplementary Fig. S6 ). Emicizumab slightly decreased thrombin peak height in high concentrations of emicizumab under high TF concentration triggering condition, and slightly extended lag time under low TF concentration triggering condition.

These properties of emicizumab would eventually enhance the parameters in these thrombin generation assay conditions, and therefore would decrease the robustness to assess the effects of emicizumab on the action of AT and TFPI. Anti-FIXa and anti-FX monospecific one-armed antibodies derived from emicizumab do not possess FVIIIa cofactor function on coagulation; therefore, we consider that these one-armed antibodies are more preferable to be used in this study.

Although the add-on effects of emicizumab on thrombin peak height were not observed in normal plasma, as it would reach a saturation state ( Supplementary Fig. S5 ), in the experiment of AT- or TFPI-depleted plasma, we assessed anti-FIXa and anti-FX monospecific one-armed antibodies also in the condition that thrombin peak heights were not reached to saturation state so that it is possible to assess changes of thrombin peak height ( Figs. 3 and 4 ).

We cannot exclude all of the effects of anti-FIXa and anti-FX monospecific one-armed antibodies on thrombin generation parameters; however, we think that we could evaluate the effect of one-armed antibodies that they do not inhibit the action of AT or TFPI because these one-armed antibodies showed almost same thrombin generation parameters compared with the antibody-absent control group ( Figs. 3 and 4 ). The presence of 1,000 nM anti-FX monospecific one-armed antibody seems to decrease peak height slightly only in extrinsic triggers ( Fig. 4D ). This one-armed antibodies also tended to exhibit extended lag time compared with the control ( Supplementary Fig. S4B ). At the concentration of 1,000 nM emicizumab, the *K*_D_ -based simulation predicted that 32% of FX would form complex with emicizumab. 13 In the assay system, we assumed that such a kind of complex formation, which would predictively occupy around 30% of FX, slightly affected the strength of triggering coagulation cascade by TF.

Heparin-like molecules, heparin sulfate proteoglycans, are closely associated with endothelial cells and enhance the action of circulating antithrombin. In chromogenic assays for FIXa and FXa activities, we examined the influence of heparin in this assay, and found that emicizumab did not interfere with the inhibitory effect of AT on FIXa or FXa in the presence of heparin ( Fig. 1B , D ). These results suggest that emicizumab would not inhibit the action of sole AT and also AT which forms complex with heparan sulfate.

The inhibition of FXa by TFPI is a biphasic, slow tight-binding mechanism. 17 In the first step, TFPI and FXa rapidly form a loose binary complex, and slow rearrangement of this initial complex results to a tight FXa–TFPI complex. In the enzymatic assay, we measured the final absorbance after the chromogenic substrate added, which reflects the second step of the inhibition of FXa by TFPI ( Fig. 2 ). In the thrombin generation assay, which monitors the formation of thrombin with time, it reflects both of the first and second steps of the inhibition of FXa by TFPI ( Fig. 4 , Supplementary Fig. S4E , F ). Our data suggest that emicizumab did not interfere with both of the steps of the inhibition of FXa by TFPI. We used C-terminus truncated TFPI in this study, which does not contain the C-terminus region that interacts with cell surfaces; however, it contains all Kunitz-type domains including the K2 domain which binds to FXa. We confirmed that emicizumab did not interfere with the full-length TFPI activity on FXa, which result was consistent with the result from C-terminus truncated TFPI ( Fig. 2 , Supplementary Fig. S2 ); therefore, we speculate that it is possible to evaluate the effect of emicizumab on TFPI by using C-terminus truncated TFPI to a certain degree at least.

In coagulation feedback system, activated protein C (APC) is also known as an important factor in addition to AT and TFPI. Unlike FVIIIa, emicizumab is not inactivated by APC. However, coagulation reaction enhanced by emicizumab could be downregulated through inactivation of FVa by APC. 18 It is shown that APC would be able to perform its coagulation feedback function even in the presence of emicizumab. 18 Taken together, it is suggested that coagulation feedback by AT, TFPI, and APC would work sufficiently in the presence of emicizumab. Therefore, contributions of these factors should be taken into account to understand the whole picture of coagulation process in the presence of emicizumab in clinical settings.

In conclusion, we demonstrated that emicizumab exerts its action without perturbing AT or TFPI, and thus should not alter the coagulant/anticoagulant balance related to AT and TFPI. These basic findings are important, for example, when considering the meanings of coagulation-related biomarkers in the presence of emicizumab.
