# Supplementary material for: Emicizumab, A Bispecific Antibody to Factors IX/IXa and X/Xa, Does Not Interfere with Antithrombin or TFPI Activity In Vitro
Source: TH Open. 2018 Mar 16;2(1):e96–e103. doi: 10.1055/s-0038-1636538 (PMC6524866; doi:10.1055/s-0038-1636538)
Supplement: Supplementary file 1 — Supplementary Figures [file 10-1055-s-0038-1636538-s170019.pdf]

Materials and Methods for Supplementary Data

Chromogenic Assays for FIXa and FXa Activities

To evaluate FIXa activity, we conducted an enzymatic assay consisting of various concentrations of human FIXa and 4 µM phospholipids in Tris-buffered saline (50 mM Tris, 150 mM NaCl) containing 1 mM $CaCl_2$ and 0.1% (w/v) bovine serum albumin (BSA), and incubated for 30 minutes at room temperature. After adding a chromogenic substrate specific to FIXa (Spectrozyme FIXa; Sekisui Diagnostics), we measured absorbance at 405 nm to determine FIXa activity.

To evaluate FXa activity, we conducted an enzymatic assay consisting of various concentrations of human FXa and 4 µM phospholipids in Tris-buffered saline containing 1 mM $CaCl_2$ and 0.1% (w/v) BSA, and incubated for 30 minutes at room temperature. After adding a chromogenic substrate specific to FXa (S-2222; Sekisui Diagnostics), we measured absorbance at 405 nm to determine FXa activity.

To evaluate FXa inhibition, we conducted an enzymatic assay consisting of 1.25 nM human FXa, 4 µM phospholipids, various concentrations of full-length recombinant human tissue factor pathway inhibitor (TFPI; Sekisui Diagnostics), and emicizumab in Tris-buffered saline containing 1 mM $CaCl_2$ and 0.1% (w/v) BSA, and incubated for 30 minutes at room temperature. After adding a chromogenic substrate specific to FXa (S-2222; Sekisui Diagnostics), we measured absorbance at 405 nm to determine FXa activity.

Thrombin Generation Assay

Thrombin generation assays were performed with standard equipment and CT using a 96-well plate fluorometer (Thermo Fisher Scientific) equipped with analyzing software (Thrombinoscope BV, Maastricht, the Netherlands). Thrombin generation was initiated with an intrinsic pathway triggering solution containing 0.04 nM human FXIa and 20 µM phospholipids in Tris-buffered saline solution containing 0.1% (w/v) BSA, or extrinsic pathway triggering solutions (PPP-Reagent HIGH, PPP-Reagent LOW; Thrombinoscope BV) in normal plasma (Sysmex, Kobe, Japan) or congenital FVIII-deficient plasma (George King Bio-Medical, Overland Park, Kansas, United States) with various concentrations of emicizumab in Tris-buffered saline solution containing 0.1% (w/v) BSA. To initiate the reaction, 20 µL of FluCa reagent prepared from a FluCa kit (Thrombinoscope BV) was dispensed by the instrument as programmed. We analyzed the thrombograms, peak height, and lag time by the instrument's software.

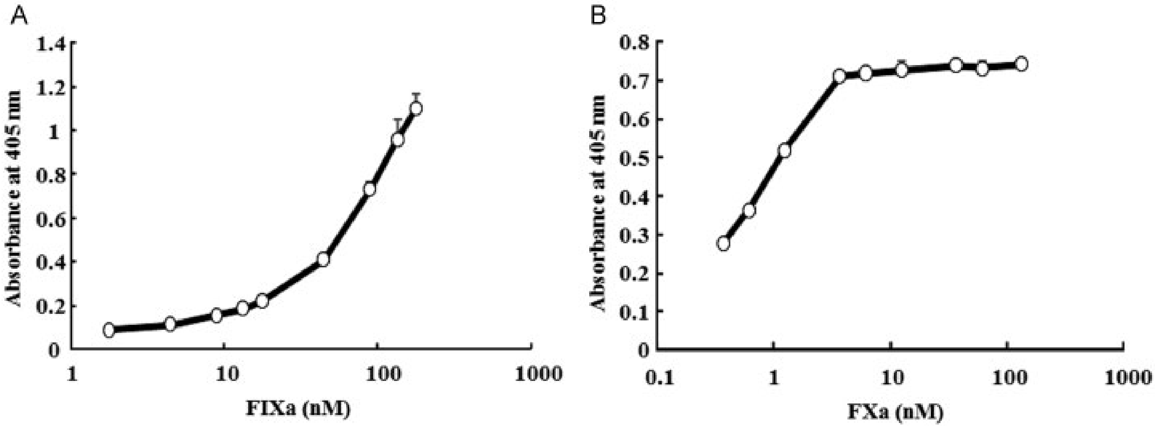

Fig. S1 Effects of FIXa and FXa concentration in FIXa and FXa enzymatic assays. (**A**) Various concentrations of FIXa assessed in FIXa enzymatic assay. (**B**) Various concentrations of FXa assessed in FXa enzymatic assay. Data are expressed as mean ± SD ($n = 3$). The bars depicting SD are shorter than the height of the symbols.

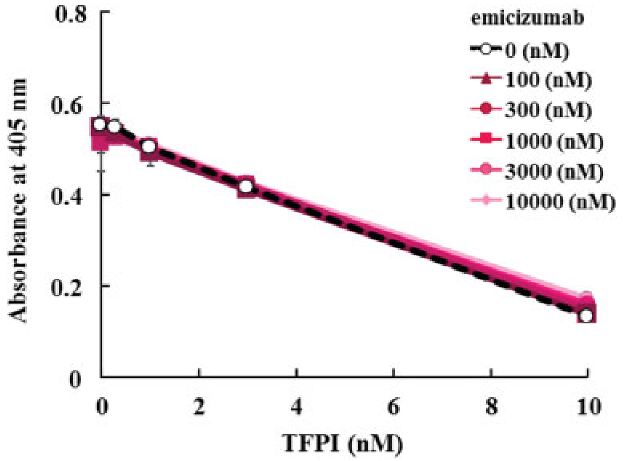

Fig. S2 Effect of emicizumab on the action of full-length TFPI on FXa in enzymatic assays. Emicizumab's effect on full-length TFPI action on FXa in an FXa inhibition assay. Data are expressed as mean ± SD ($n = 3$). The bars depicting SD are shorter than the height of the symbols. The symbols for the groups with higher concentrations of emicizumab are hidden behind the symbols for the other groups.

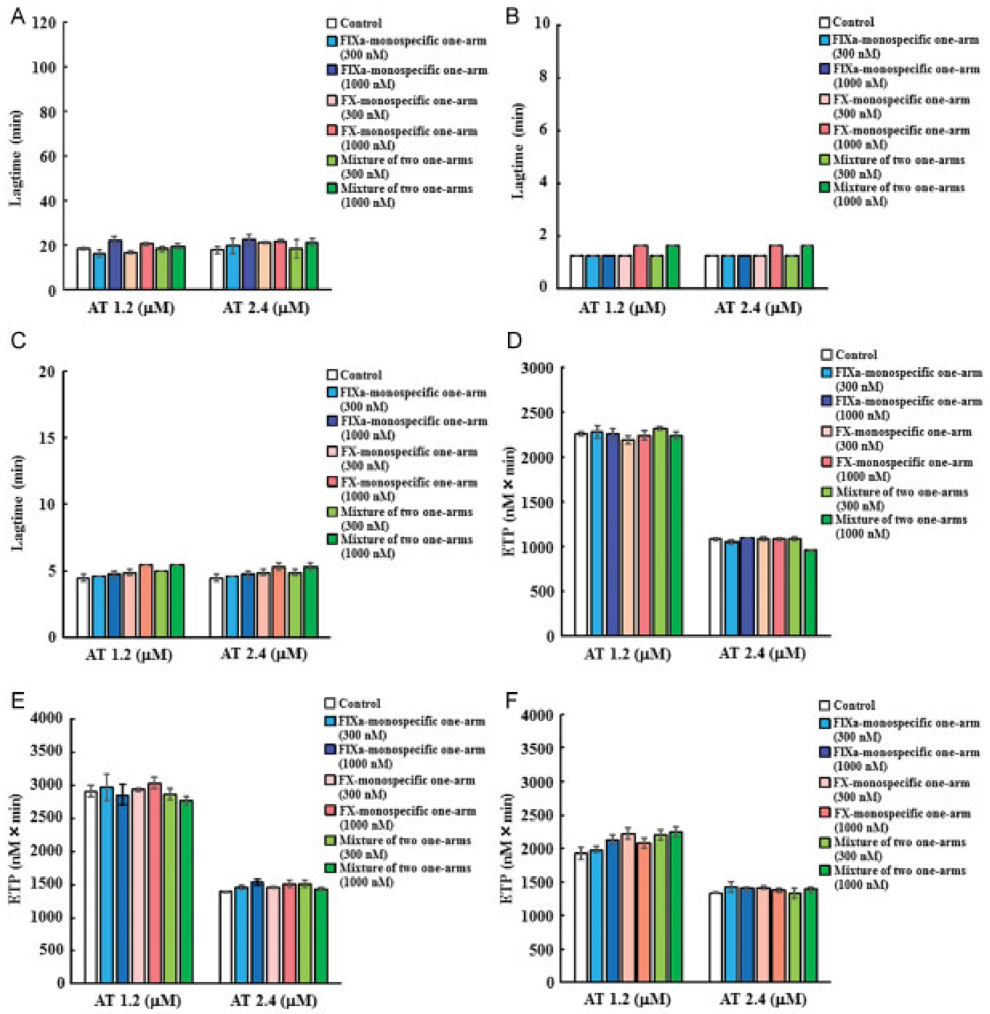

Fig. S3 Effects of AT and FIXa and FX monospecific one-armed antibodies on thrombin generation in AT-depleted plasma. (**A–F**) Effects of various concentrations of anti-FIXa and anti-FX monospecific one-armed antibodies, and mixtures of the two one-armed antibodies on the thrombin generation parameter lag time and ETP in AT-depleted plasma triggered by (**A**, **D**) FXIa, (**B**, **E**) high concentration of TF, and (**C**, **F**) low concentration of TF in the presence of 1.2 or 2.4 µM of AT. Data are expressed as mean ± SD ($n = 3$).

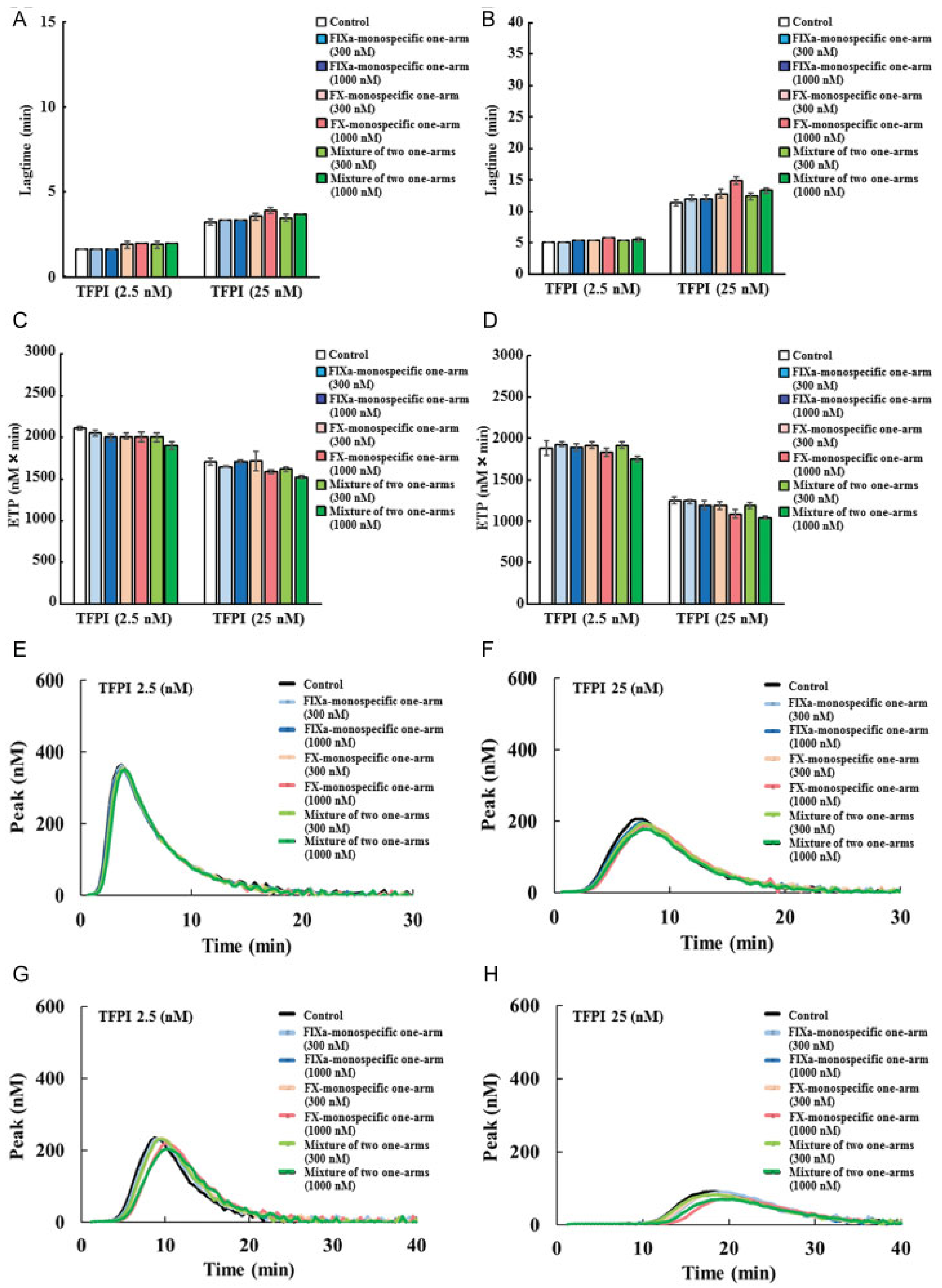

Fig. S4 Effects of TFPI and anti-FIXa and anti-FX one-armed antibodies on thrombin generation in TFPI-depleted plasma. (**A–H**) Effects of various concentrations of anti-FIXa and anti-FX monospecific one-armed antibodies, and mixtures of the two one-armed antibodies on the thrombin generation parameter lag time and ETP in TFPI-depleted plasma triggered by (**A**, **C**, **E**, **G**) high concentrations of TF and (**B**, **D**, **F**, **H**) low concentrations of TF in the presence of 2.5 and 25 nM of TFPI. (**A–D**) Data are expressed as mean ± SD ($n = 3$). (**E–H**) Data were collected in triplicate and representative thrombograms are shown.

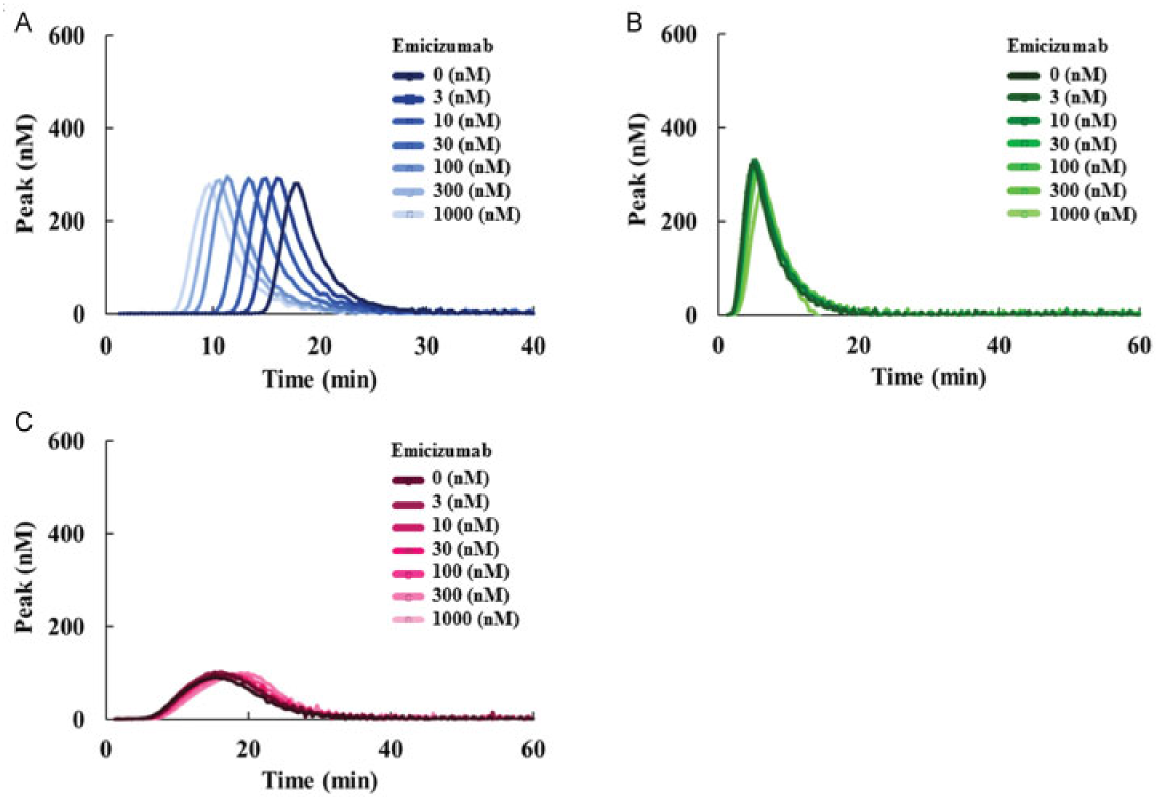

Fig. S5 Effect of emicizumab on thrombin generation in normal plasma. Effects of various concentrations of emicizumab on the thrombin generation in normal plasma triggered by (**A**) FXIa, (**B**) high concentration of TF, and (**C**) low concentration of TF. Data were collected in triplicate and representative thrombograms are shown.

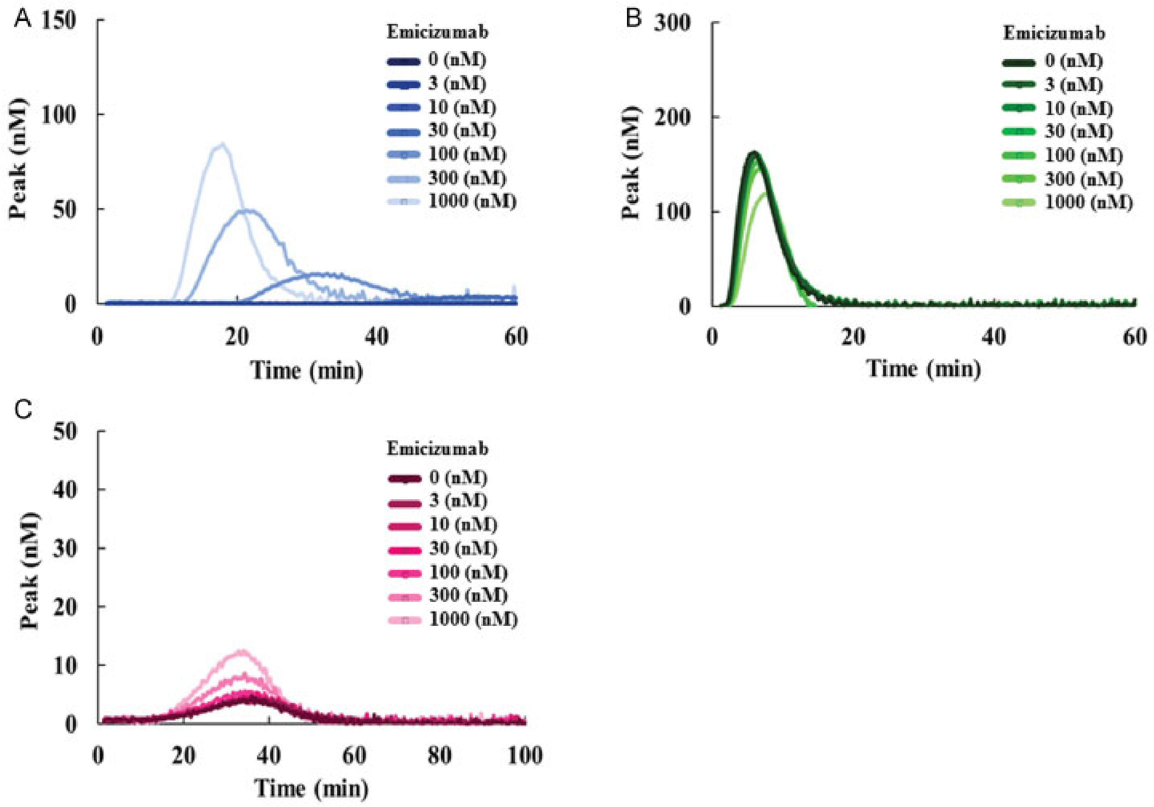

Fig. S6 Effect of emicizumab on thrombin generation in FVIII-deficient plasma. Effects of various concentrations of emicizumab on the thrombin generation in FVIII-deficient plasma triggered by (**A**) FXIa, (**B**) high concentration of TF, and (**C**) low concentration of TF. Data were collected in triplicate and representative thrombograms are shown.
